# Organometallic vs organic photoredox catalysts for photocuring reactions in the visible region

**DOI:** 10.3762/bjoc.14.282

**Published:** 2018-12-12

**Authors:** Aude-Héloise Bonardi, Frédéric Dumur, Guillaume Noirbent, Jacques Lalevée, Didier Gigmes

**Affiliations:** 1 Université de Haute-Alsace, CNRS, IS2M UMR 7361, F-68100 Mulhouse, France; 2 Université de Strasbourg, France; 3 Aix Marseille Univ, CNRS, ICR UMR 7273, F-13397 Marseille, France

**Keywords:** photoinitiator, photopolymerization, photoredox catalysis, photoredox catalyst

## Abstract

Recent progresses achieved in terms of synthetic procedures allow now the access to polymers of well-defined composition, molecular weight and architecture. Thanks to these recent progresses in polymer engineering, the scope of applications of polymers is far wider than that of any other class of material, ranging from adhesives, coatings, packaging materials, inks, paints, optics, 3D printing, microelectronics or textiles. From a synthetic viewpoint, photoredox catalysis, originally developed for organic chemistry, has recently been applied to the polymer synthesis, constituting a major breakthrough in polymer chemistry. Thanks to the development of photoredox catalysts of polymerization, a drastic reduction of the amount of photoinitiators could be achieved, addressing the toxicity and the extractability issues; high performance initiating abilities are still obtained due to the catalytic approach which regenerates the catalyst. As it is a fast-growing field, this review will be mainly focused on an overview of the recent advances concerning the development of organic and organometallic photoredox catalysts for the photoreticulation of multifunctional monomers for a rapid and efficient access to 3D polymer networks.

## Introduction

Photopolymerization reactions are now widely used both in industry and in academic laboratories. These processes usually lead to the transformation of a liquid resin in a 3D solid polymer upon light exposure. These photochemical processes offer potential advantages compared to thermal polymerization. First, it is a greener technology, i.e., the system does not need to be heated and no (or low content of) volatile organic compounds are released. Secondly, mild conditions can be employed. It is now possible to perform photopolymerizations upon soft irradiation conditions with, for example, household light bulbs, LED light, low intensity lasers and even sunlight [1–4]. The first reason is the low cost and infiniteness character of light (more particularly when using visible light). The “on-off” aspect of a lamp offers good possibilities of external regulator of the reaction. Another advantage is the spatial control, i.e., the reaction only occurs in the light-irradiated areas. For all these advantages, photopolymerization reactions are already encountered for applications in a lot of sectors such as coatings, adhesives, paints, inks, composites, 3D-printing, dentistry, data storage ... [5–7].

## Review

### 1 Photopolymerization processes and uses of photocatalysts (PCs)

Traditionally, polymer manufacturing is made through thermal curing. However, this route has many limitations: these processes are usually slow, expensive by requiring high temperature and high energy and release solvent (VOC). As an alternative of thermal polymerizations, polymerization upon light irradiation offers a good alternative. In both cases, polymerization occurs by the action of an initiating system in a monomer blend. In the case of photopolymerization, a photoinitiating system (PIs) is required to convert light in initiating species. Many factors affect the photopolymerization kinetics. However, the PIs is the most important key factor and that’s why enhancing its properties have drawn many interest in the past few decades. The development of photoredox catalysts is one of the major advances.

#### 1.1 Photoinitiating system (PIs)

Photoinitiated polymerization processes are polymerizations initiated by light irradiation. For that, a photoinitiator (PI) or a photoinitiating system (PIs) is mixed with the monomer or a monomer blend. The PI is a component which absorbs light and initiates polymerization alone whereas a photoinitiating system comprises different compounds [8–10]. Under irradiation, PI or PIs generate active species: free radicals and/or ions and/or acid. When the active species are produced, a wide range of monomers can be polymerized via a free radical or a cationic mechanism (anionic mechanisms are still rare) [8]. A PI can also be used in combination with a photosensitizer (PS) to extend the spectral sensitivity to longer wavelengths. The development of new photoinitiating systems has been the subject of many researches in the last decades [11–12].

For a PIs to be efficient, it requires several properties:

i) Excellent light absorption (meaning high molar extinction coefficients) in the region of the emission spectrum of the irradiation source.

ii) Appropriated excited state energy and redox potentials to interact with additives [13].

Parallel to this, the environmental issues impose the use of new polymerization methods that are safer for the manipulator, contribute to lower the amount of released volatile organic compounds and can reduce the energy consumption used to produce the polymers. At present, most of the photoinitiating systems in use in the industry required high light intensity, the emission of these lamps being mainly centered in the UV range. To create safer photoinitiating systems, photoinitiators exhibiting a strong absorption in the near UV or visible range with high molar extinction coefficients are actively researched. As far as the extractability of the photoinitiators and the side-products that can be formed during the polymerization process is concerned, a good strategy is to reduce the amount of photoinitiator. Thus, inspired by catalytic cycles used in organic chemistry, the development of photoredox catalysis for photopolymerization reactions has been proposed. It has emerged as a significant innovation in the field of photoinitiated polymerization. Photoredox catalysis is a new strategy to generate radicals and/or cations upon soft irradiation [14].

#### 1.2 Development of photoredox catalysts

In the field of photoinitiated polymerization, a photoredox catalyst is a photoinitiating system capable of regeneration during the polymerization reaction [14]. This regeneration is based on an oxidoreduction reaction between the light absorbing compound and suitable additives under light to induce a catalytical cycle.

As light is an inexhaustible and renewable energy, photochemistry has dealt a great interest into organic chemistry and green chemistry since the early 20th century [15]. By absorbing light, the compound reaches an electronically excited state which significantly changes the distribution of electrons in the molecule. Thus, chemical properties such as reactivity, oxidation potential or reduction potential change drastically. With appropriate donors or acceptors, electron charge transfer is possible via this excited state and redox reactions are possible. This process is called photoinduced electron transfer (PET).

**Figure 1:**
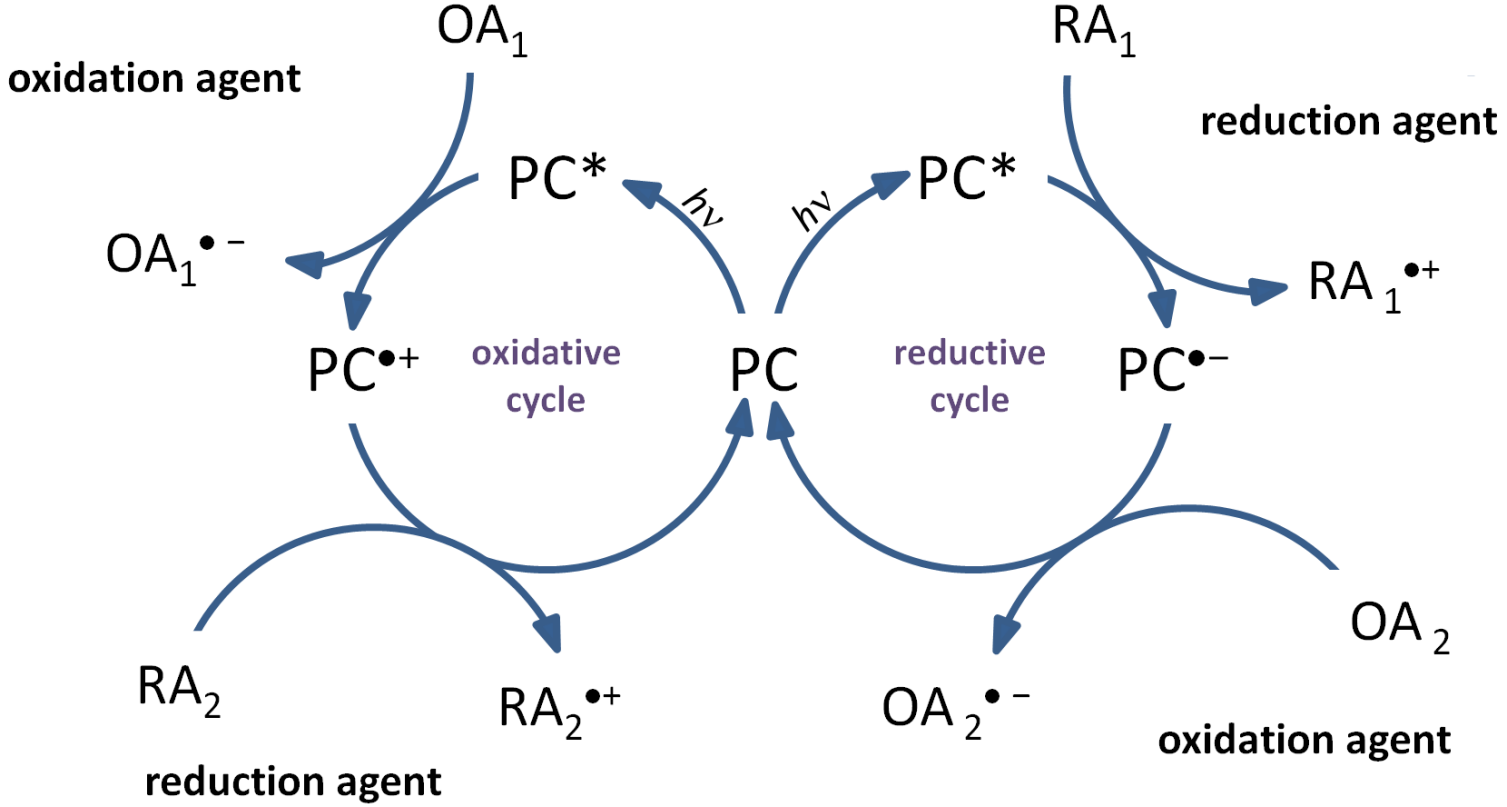
Typical oxidative and reductive cycle for a photoredox catalyst (PC).

In this context, photoredox catalysis was developed. Light is used to excite the photoredox catalyst which allows electron transfer processes with additives. Both oxidation and reduction reactions can be possible. Indeed, when the photoredox catalyst is excited, one electron moves from the HOMO (abbreviation for highest occupied molecular orbital) to the LUMO (abbreviation for lowest unoccupied molecular orbital). Thus, there is a lack of one electron in the HOMO and an electron available in the LUMO. That's why the excited photoredox catalyst is at the same time a stronger oxidant and a stronger reductant than its ground state. Therefore, the PC can react more easily with an oxidant or a reductant (see in Figure 1) [16]. By addition of another reduction or oxidation agent, the catalytic cycle can be created as illustrated in Figure 1 to regenerate the PC.

As illustrated, for an oxidative cycle, the excited photocatalyst (PC*) reacts first with an electron acceptor (also named oxidation agent, OA_1_ in Figure 1) which leads to PC^●+^ and OA_1_^●−^ . Then, PC can be regenerated with an electron donor (also named reduction agent, RA_2_ in Figure 1) by a redox reaction. In a reductive cycle, the PC* reacts first with a reduction agent (in Figure 1, RA_1_) which leads to PC^●−^ and RA_1_^●+^. Then, PC can be regenerated with an oxidation agent (OA_2_). Radicals and cations are generated in these cycles [16]. As we can observe, this process offers the possibility to regenerate the catalyst. Consequently, the amount of PC used for the photochemical transformation is added only in catalytic amount and recovered after the reaction. That’s why the definition of photocatalyst is fulfilled.

For a compound to be efficient as photoredox catalyst in the visible range, several parameters have to be fulfilled: [17]

i) The molecule should strongly absorb in the near UV or visible range with high molar extinction coefficients.

ii) The redox potentials of the excited state of photoredox catalysts must be in appropriateness to those of the additives to be incorporated into oxidative or reductive cycles (see Figure 1).

iii) The oxidation or the reduction of the photoredox catalyst should be reversible in order to be regenerated (to avoid any side reaction from PC^●+^ or PC^●−^).

iv) The excited state lifetimes of photoredox catalyst should be long enough to exhibit a high quenching efficiency with the additives, i.e., to have enough time to react with the additives.

This is frequently referred as the Golden Rules of photoredox catalysis.

Photoredox catalysis has been largely developed in organic chemistry in the last decades and already found wide applications such as in water splitting, solar energy storage, proton-coupled electron transfer or photovoltaic for example [18].

#### 1.3 Electronic transitions involved into photoredox processes

For selected photoredox catalysts, light irradiation has enough energy for the excitation of the PC from the ground state S0 to an excited stated (S1). This is usually a transition between the HOMO and the LUMO. Both can be different type of molecular orbital (MO) regarding the compound chosen and the different transitions will be presented.

**Figure 2:**
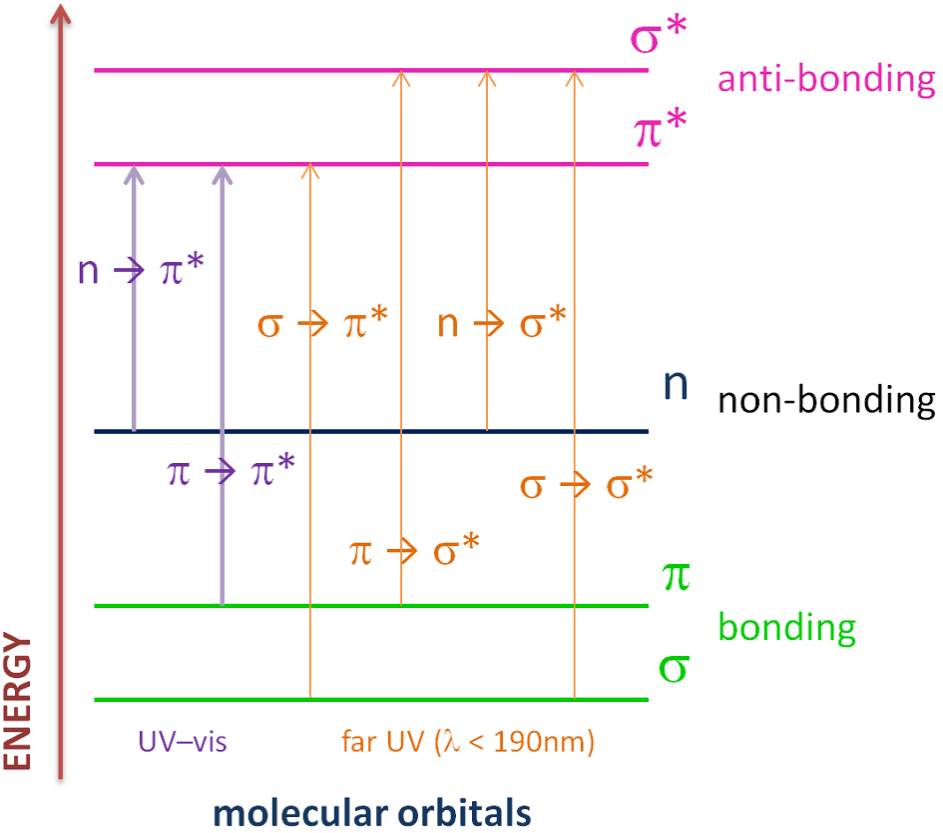
Transitions involved in absorbing species containing π, σ and n electrons.

1.3.1 n–π* transition: The n–π* transition (depicted in Figure 2) is a transition where the HOMO is a non-bonding orbital (n) and π* an anti-bonding orbital. Both orbitals have different symmetries and this transition is observed for molecules with a heteroatom such as nitrogen, oxygen or sulfur which are carrying free electron pair. Most compounds concerned by this transition usually absorbs around 300 and 380 nm and rarely up to the visible range [19].

1.3.2 π–π* transition: π referred to a bonding orbital. The π and π* molecular orbitals have generally the same symmetry which allow the π–π* transition (depicted in Figure 2).

This transition is generally observed for molecules with extended π-conjugated systems. As π and π* are more distant from each other than n and π* orbitals, the absorption is generally observed in higher wavelength than for n–π* transition. Moreover, the longer the π-system, the higher are the wavelengths needed to excite the molecule.

**1.3.3 Charge transfer transition (CT):** A charge transfer transition is mostly observed from interaction between the LUMO on an electron accepting group and the HOMO on an electron-donating group. This can be an intermolecular or intramolecular process. For an intramolecular process, this type of transition mostly concerned polarized molecules with both groups on its structure. Intermolecular charge transfer transition is observed for example with charge transfer complex formed by interaction an acceptor and a donor. The interaction between the two compounds induced a complex with a smaller energy gap between HOMO and LUMO than the energy gap between the HOMO of the acceptor and the LUMO of the donor when the two compounds are separated. This transition is generally observed with high intensity on the UV–visible spectra [20].

**Figure 3:**
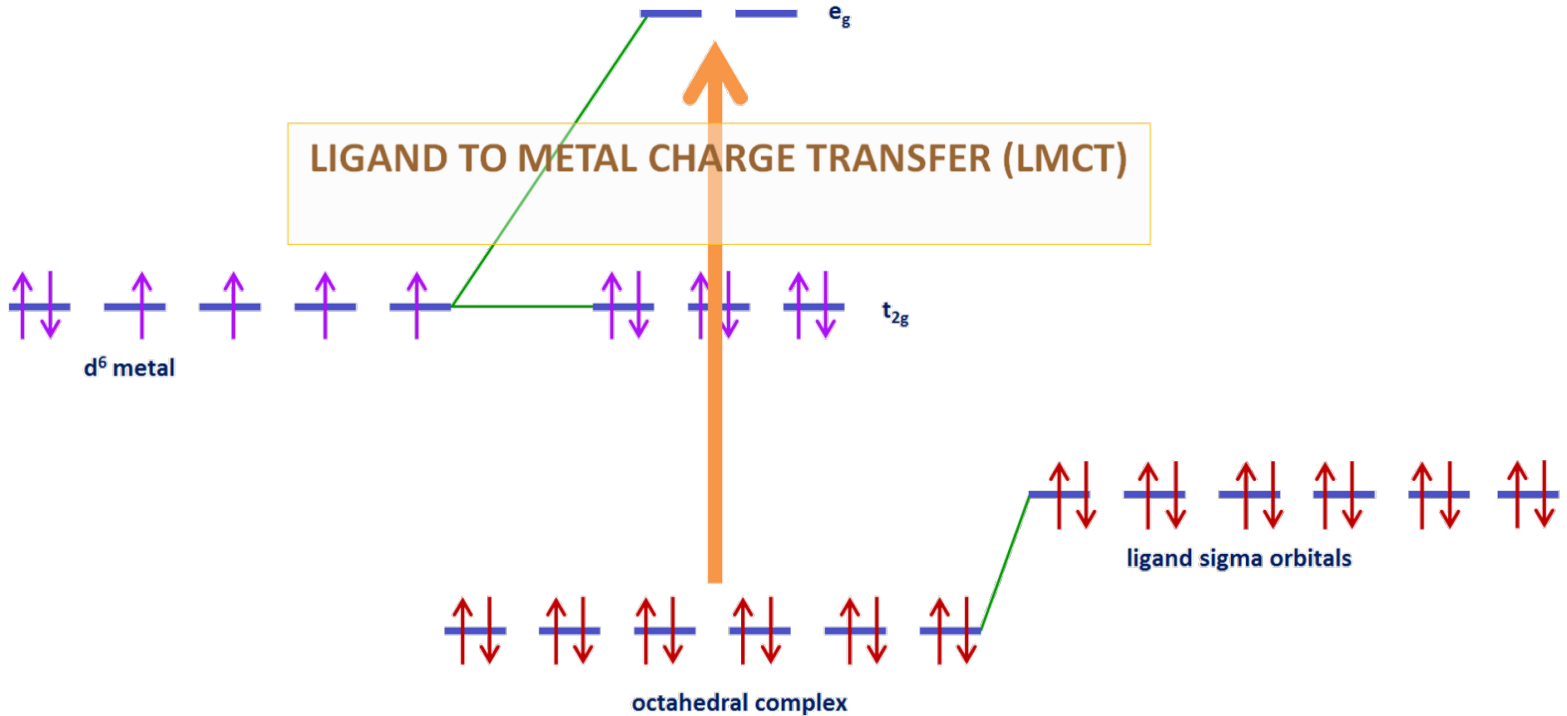
Ligand to metal charge transfer (illustrated here for a d^6^ metal complex).

**1.3.4 Ligand to metal charge transfer (LMCT):** The LMCT transition is observed for organometallic compounds for example with organic molecules as ligands. This ligand possesses σ, σ*, π, π* and n molecular orbitals [21]. When orbitals of this ligand are fully occupied, a charge transfer is possible from it to the empty or partially filled metal d-orbitals as illustrated in Figure 3. Absorption band observable are very intense.

**Figure 4:**
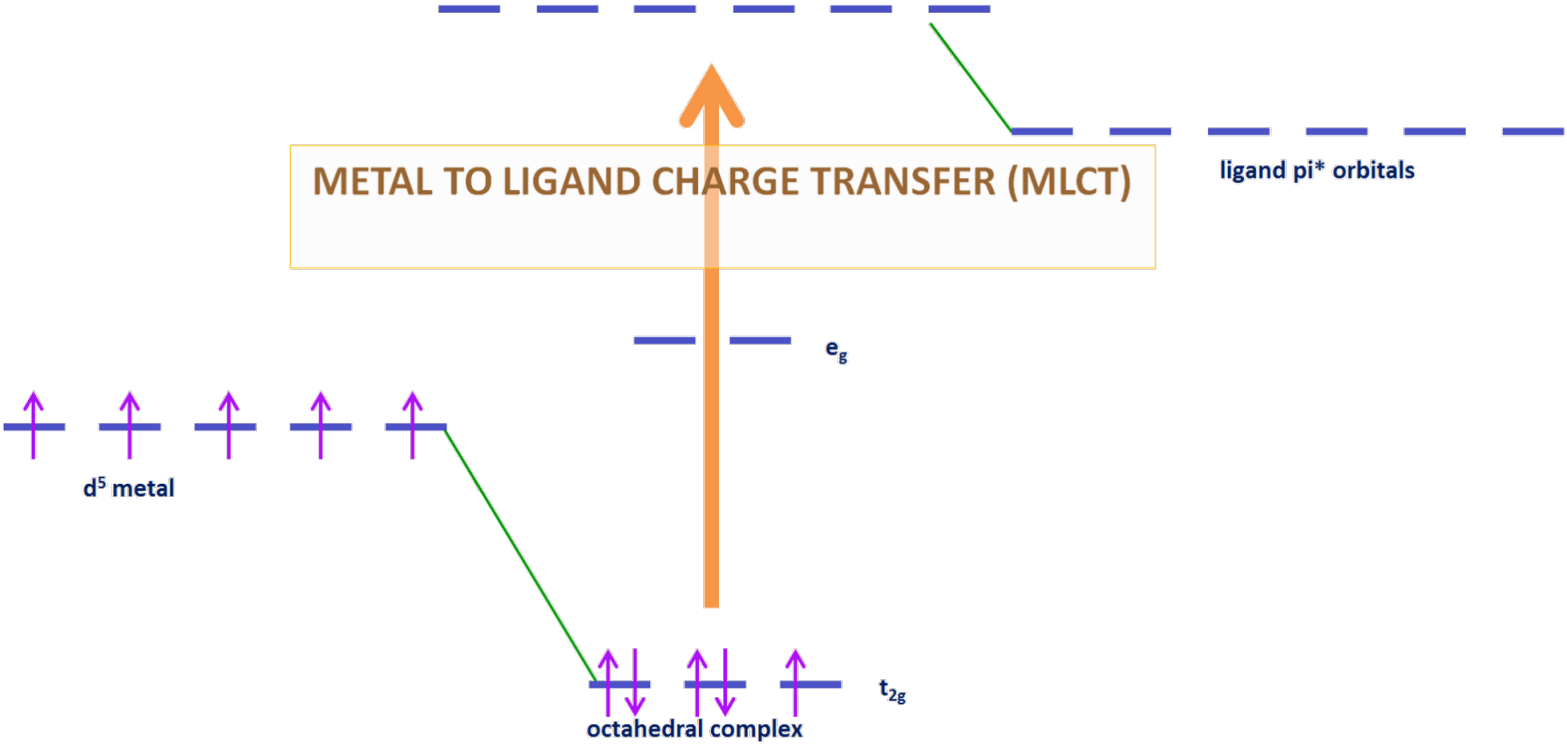
Metal to ligand charge transfer (illustrated here for a d^5^ metal complex).

**1.3.5 Metal to ligand charge transfer (MLCT):** The MLCT transition is a second type of charge transfer observed with metal complexes. More particularly, it is observed for complexes whose ligands have relatively high-energy lone pairs or in case of metal with low-lying empty orbitals. For coordination compounds with π-acceptor ligand, MLCT transition are common and can be generate through light excitation. This absorption results from the movement of an electron from the metal orbitals to the ligand π* orbitals [22]. This process is illustrated in the case of a d^5^ high spin octahedral complex in Figure 4. As for LMCT, MLCT give intense band in UV spectrum.

#### 1.4 Mechanisms in polymerization reactions

Free radical polymerization or/and cationic polymerization can be initiated by photoredox catalysis. Respectively, radicals or/and cations must be produced to initiate the polymerization. By formation of radicals, the polymerization of C=C functions such as (meth)acrylates or styrene can be initiated. With cations or acids as initiating species, epoxy monomers can be polymerized. Both types of polymerization are widely used both in academic and industrial fields. About 45% of the manufactured plastic material and 40% of synthetic rubber are produced by free radical polymerization worldwide [23].

**Scheme 1:**
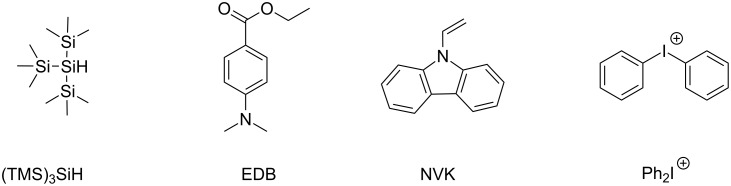
Structures of additives involved in the photoredox catalytic cycles.

In both cases, the photoredox catalyst, used as PS, absorbs the light and goes to its excited state. Then, there are two possibilities: the photoredox catalyst can react through an oxidative or a reductive cycle as presented in Figure 1. Herein, we will present four additives that can be used in combination with a photoredox catalyst to initiated photopolymerization (Scheme 1).

As reduction agent, silanes such as tris(trimethylsilyl)silane (abbreviated (TMS)_3_SiH), amines such as ethyl 4-(dimethylamino)benzoate (abbreviated EDB) or carbazole derivatives such as 9-vinylcarbazole (NVK) are presented. As oxidizing agent, it is possible to use a iodonium salt such as diphenyliodonium (Ph_2_I^+^).

With these additives, three systems involving catalytic cycles for photopolymerization are presented: [14,23–24]

- PC/Ph_2_I^+^/(TMS)_3_SiH
- PC/Ph_2_I^+^/NVK
- PC/Ph_2_I^+^/EDB

**Figure 5:**
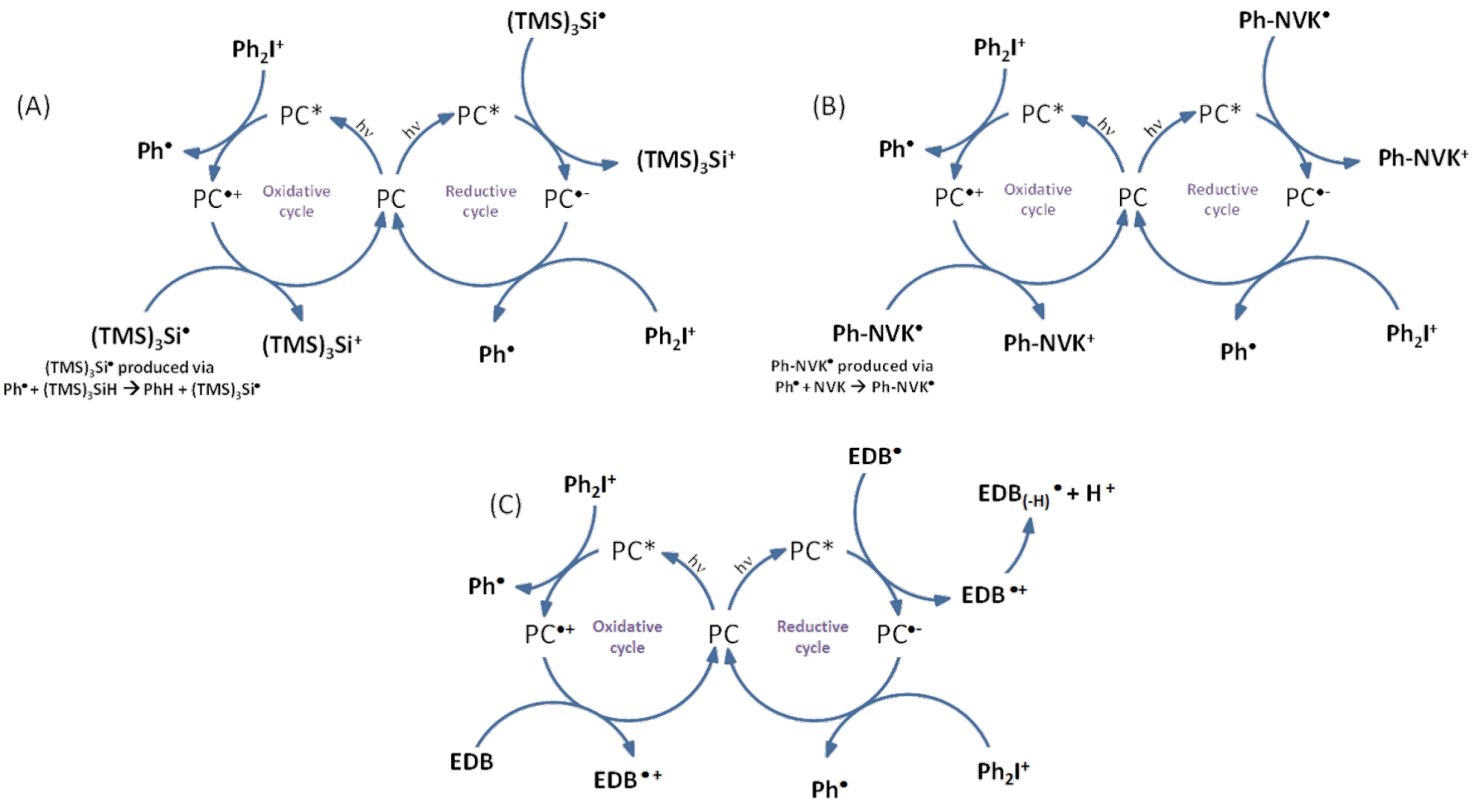
Catalytic cycles involved with iodonium salt and (A) (TMS)_3_SiH, (B) NVK and (C) EDB.

With these three systems, both free radical and cationic polymerizations are possible. The chemical mechanisms for these different systems are depicted in Figure 5.

With appropriated photoredox catalysts, formations of interpenetrated polymer networks (IPN) are also mentioned. For the three systems proposed above, formation of aryl radicals is observed. These radicals are able to initiate the free radical polymerization of (meth)acrylates [1]. In the photocatalytic cycle (Figure 5C), EDB_(−H)_^•^ radicals are also produced and able to initiate the free radical polymerization. Concerning the cationic polymerization, initiating cations are also produced in the three systems proposed, e.g., in the catalytic cycle (Figure 5A). Thanks to the low ionization potential of the silyl radicals (TMS)_3_Si^•^, the generation of silylium cations is possible. These cations have been described in the literature for initiation of ring-opening polymerization processes [25]. Cations Ph-NVK^+^ produced in catalytic cycle (Figure 5B) have also been well noted in the literature as highly reactive structures [26–27]. Amines, such as EDB presented in photoredox catalytic cycle (Figure 5C), are also well mentioned as efficient co-initiators for free-radical-promoted cationic polymerizations [1,28–29].

In Part 2, a photoredox catalyst useable in such a photocatalytic system will be presented. To be involved properly into the photocatalytical cycle, photoredox catalysts must be chosen with suitable redox potential to perform an oxidation or a reduction with other additives presented above. Thus, redox potentials of additives are resumed in Table 1.

**Table 1:** Redox properties of additives [1,30–31].

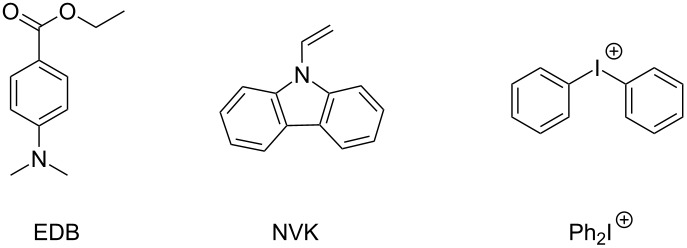			

additives	reaction	redox potential associated	references

Ph_2_I^+^	reduction	−0.2 V	1
NVK	oxidation	1.17 V	30
EDB	oxidation	1.1 V	31

Indeed, additives and photoredox catalysts are involved into redox mechanisms. From a single or triplet state of the photoredox catalyst, an electron is transferred. According to Rehm–Weller, an electron can be transferred from the electron donor to the electron acceptor in the excited state if the free energy change ∆*G*_et_ is negative. ∆*G*_et_ can be calculated from the equation:

[1] $$\Delta G_{\text{et}}=E_{\text{ox}}–E_{\text{red}}–E*\;+\;C$$

where *E*_ox_ is the oxidation potential of the electron donor, *E*_red_ the reduction potential of the electron acceptor, E* the excited state energy level and C the coulombic term for the initially formed ion pair (if there are ions in solution). For polar solvent, C is neglected.

(TMS)_3_SiH is not presented in Table 1 because the driving force of its reactivity is more its bond dissociation energy (BDE) than its redox potential. Indeed, this compound obeys to a pure hydrogen transfer mechanism. This corresponds to a hydrogen transfer from (TMS)_3_SiH to the triplet excited state of the PC. Thus, to react, the PC must have a lifetime of its triplet excited state that is long enough. The bond dissociation energy of the Si–H of (TMS)_3_SiH has been calculated: 79.8 kcal/mol (methods of calculation optimized at the B3LYP/6-31G* level as referred in [32]). Polymerization performances of photoredox catalysts given in example in the present review will be presented in Part 3.

### 2 The different classes of photoredox catalysts (PCs)

**Scheme 2:**
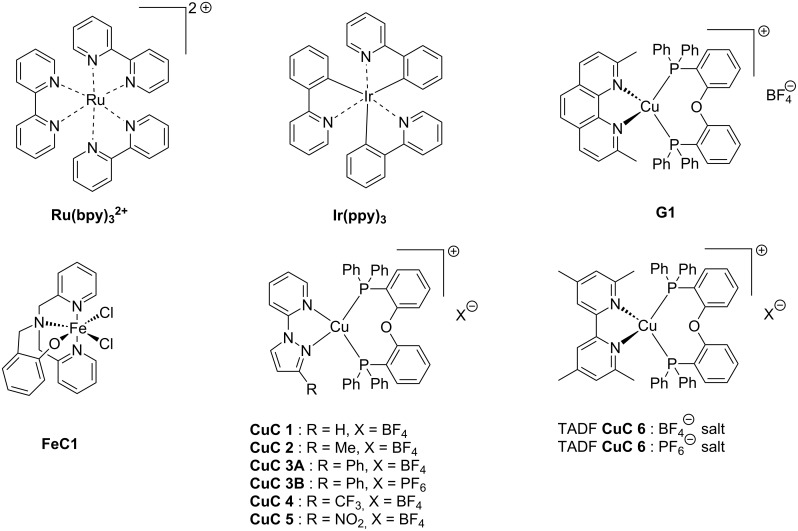
Structures of photoredox metal-based catalysts.

The main characteristics of the different classes of photoredox catalysts will be given below. If historically, photoredox catalysts were based on metals, but recent developments have promoted the emergence of metal-free catalysts that could in the future discard those based on metals, notably due to cost and environmental issues. Both categories of photoredox catalysts (PCs) will be described in this part and a series of structures is given in Scheme 2.

#### 2.1 Metal-based photoredox catalysts

The first photoredox catalysts to emerge were the metal-based complexes. Indeed, metal complexes, also named coordination compounds, have been recognized in the photochemistry field since the second half of the last century [33–35]. However, these compounds are still the subject of extensive researches [36]. Coordination compounds have interesting properties for photochemical reactions. First, by absorption of visible light, transitions described in part 1.3 can be observed. Thus, the complex goes from its ground state to one electronically excited state which produced reactive species. Most of the transitions observed for this complex are LMCT and MLCT (respectively, described in 1.3.4 and 1.3.5). After electron transfer, the metal complex is promoted to an oxidation or reduction state. With well adapted redox potentials and relatively long-lived excited states, these metal-based complexes can be used into photoredox catalysis with suitable oxidation or reductive agents [37–39].

**2.1.1 The first generation of metal-based PCs: ruthenium and iridium complexes:** The first generation of coordination compounds used for the photoredox catalysis comprises ruthenium and iridium complexes. These two types of metal-based complexes have been substantially developed in the last 40 years for different photochemical applications [40–42] and found wide applications in solar cells [43] or OLEDs (organic light-emitting devices) fabrication [44] and more recently in free radical polymerization [45]. Both complex families can react through oxidative or reductive pathways (Figure 1) depending on the other chemical compounds mixed with them (see below in Part 3 for the polymerization initiated by photoredox catalysis). The light absorption properties of these complexes can be tuned by modification of the structure and more specially by choosing the appropriate ligands. Photochemical reactions can occur upon different light expositions with wavelengths ranging from UV to red light. Low light intensity can be used too, such as the one delivered by household LED bulbs [14]. **Ru(bpy)_3_^2+^** is probably the most studied Ru-based photoredox catalyst (abbreviation for ruthenium tris(2,2'-bipyridyl) dichloride; depicted in Scheme 2). The photochemical properties of this complex, commercially available, are gathered in Table 2.

**Table 2:** Characteristics of **Ru(bpy)_3_^2+^** [45–48].

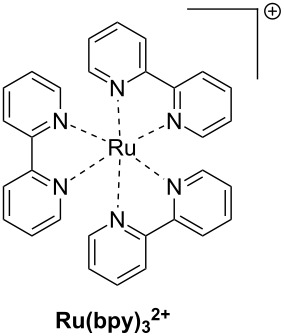		

		references

appearance	red solid	
transitions observed	MLCT transition (strong absorption around 450 nm) d–d transition (weak absorption around 350 nm) ligand centered π–π* transition (285 nm)	[45]
nature of the excited state	triplet	[46]
excited state lifetime	1100 ns	[47]
oxidation potentials reduction potentials	*E*_1/2_^ox^ = +1.29 V; *E*_1/2_^+II/I^ = +0.77 V *E*_1/2_^III/+II^ = −0.81 V; *E*_1/2_^red^ = −1.33 V	[48]

**Scheme 3:**
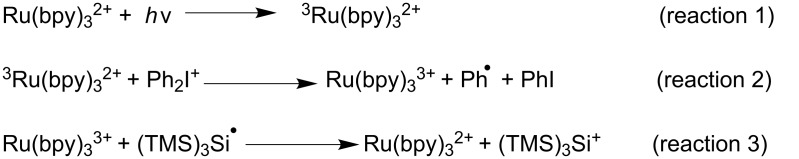
Photocatalytical cycle for the Ru complex.

By irradiation of the ruthenium complex, there is a formation of a triplet excited state through metal to ligand charge transfer (Scheme 3, reaction 1). As described in Table 2, the irradiation must be around 450 nm. Thus, as the triplet excited state is long enough and thanks to the values of oxidation potentials, a single electron transfer (SET) to the iodonium salt occurred (Scheme 3, reaction 2) [46]. The formation of phenyl radicals is observed. Another product of reaction 2 is Ru(bpy)_3_^3+^. This species is able to react with (TMS)_3_Si**^●^** to regenerate **Ru(bpy)_3_^2+^** (reaction 3). Thus, the photocatalytical cycle is observed.

To conclude, oxidative and reductive photoredox cycles can be observed with Ru(bpy)_3_. Other ruthenium complexes which can be used into photocatalytical cycles have been described in the literature and more particularly with other type of ligands. Modification of the ligands has an influence on the redox potentials and the lifetime of the excited states [40]. The more the ligand has an electron-donating behavior, the easier is the oxidation of the metal center. For example, adding methyl substituents to the bipyridine ligands of the **Ru(bpy)_3_^2+^** complex, the reduction potential shifts from −1.33 V to −1.45 V [49].

Redox potentials of the complex can also be tuned by changing the metal. For example, smaller Stokes shifts are observed using iridium rather than ruthenium metal. That’s why Ir-based complexes have also been widely described in the literature as metal-based photoredox catalysts [19]. As an example, **Ir(ppy)_3_** (abbreviation for tris(2-phenylpyridine)iridium) has been chosen and is depicted in Scheme 2. This metal complex is also commercially available. **Ir(ppy)_3_** exhibits a MLCT transition in the near UV–visible range. The photochemical properties of this complex are gathered in Table 3.

**Table 3:** Characteristics of **Ir(ppy)_3_** [40,50–51]:

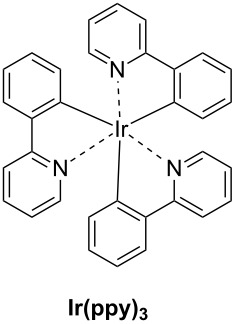		

		references

appearance	yellow to orange solid	
transitions observed	MLCT transition (strong absorption around 375 nm) d–d transition (weak absorption around 278 nm) ligand centered π–π* transition (230 nm)	[40]
nature of the excited state	triplet	[50]
excited state lifetime	1900 ns	[50]
oxidation potentials reduction potentials	*E*_1/2_^ox^ = +0.78 V; *E*_1/2_^+II/I^ = +0.31 V *E*_1/2_^III/+II^ = −1.73 V; *E*_1/2_^red^ = −2.20 V	[51]

Interestingly, it is also possible to tune both the light absorption and redox properties through well selected ligands [52]. This Ir-based complex reacts in the photoredox catalytical cycle with iodonium salt and (TMS)_3_SiH as the Ru-based complex in reactions 1, 2 and 3. Moreover, Iridium complexes can be interesting for applications in ring-opening photopolymerization initiation as shown in [53] (see Part 3). For this purpose, the Ir complex was more interesting than the Ru one because of the longer excited state lifetime, lower oxidation potential leading to higher interaction rate constants with additives used for ring-opening photopolymerization (e.g., iodonium salt). Thus, promising photoinitiating systems using Ir complexes have been proposed for polymerization under household fluorescence bulbs and even under sun radiation (useful for development of green technologies) [54]. Despite the really interesting properties of Ru and Ir complexes, they have also strong drawbacks limiting their applications to photosensitive systems. First of all, this first generation of complexes is very expensive and can be pretty hard to synthesize. Moreover, the complexes can be toxic [55]. Therefore, it was essential to develop new photoredox catalysts based on low-cost metals such as copper complexes or iron complexes which will be described in detail in the paragraph below.

**2.1.2 The second generation of metal-based PCs: copper and iron complexes:** Due to their earth-abundant property, iron and copper have received increasing attention and development of Fe and Cu complexes as highly efficient photoredox catalysis has been the subject of many studies [55–58]. These complexes have been identified as really efficient for developing low-cost electroluminescent devices or light-mediated reaction such as polymerization upon near UV or visible light applications (see Part 3).

The development of efficient photoluminescent copper complexes is possible by choosing appropriate ligands. The fine tunings of both redox potentials and visible light absorption properties are also possible [59]. Copper complexes can show really interesting properties for photoredox catalyst applications. Some of them exhibit high emission quantum yields, long excited-state lifetimes and high oxidation potentials adequate for photoredox catalysis [59–62].

One example of highly efficient copper complex developed for photoredox applications is [Cu(neo)(DPEphos)]BF_4_ also named **G1** (depicted in Scheme 2). The synthesis of this complex is detailed in references [63] and [64]. The photochemical properties of **G1** are gathered in Table 4.

**Table 4:** Characteristics of **G1** [63–65].

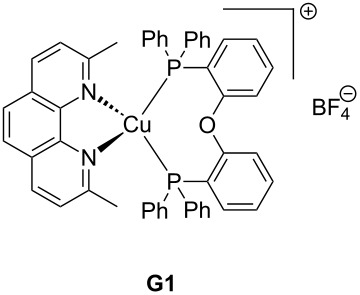		

		references

appearance	yellow solid	
transition observed	MLCT transition (strong absorption at 380 nm) more intense intraligand transitions appear at shorter wavelength	[63]
nature of the excited state	triplet	[63]
excited state lifetime	3000 ns	[64]
oxidation potential	*E*_1/2_^ox^ = +1.35 V	[65]

The multidentate ligands confer to the complex MLCT possibilities like the Ru complexes or Ir complexes described above. This transition is observed after a strong absorption of the complex centered at 380 nm (Table 4). Thus, it can react through a redox cycle with similar reaction than the Ru-based complexes under irradiation by different visible light such as a halogen lamp, laser diodes (405 and 457 nm) or LEDs (405 and 455 nm) [63].

Iron oxide photoredox catalysts have been also developed but not for polymer applications [65]. Iron oxide can offer the same advantages than TiO_2_ but with a lower gap between its HOMO and its LUMO which enable visible light excitation and thus, wide applications in heterogeneous photocatalysis for example. Therefore, a large series of iron complexes were also reported as photoredox catalysts. The photosensitivity of this class of transition metal has been recognized since the middle of the last century. Intense absorption bands of these complexes are located in the ultraviolet range and are related to a charge-transfer transition [33]. An example of an iron complex photoredox catalyst **FeC1** is given in Scheme 2 and the associated photochemical properties gathered in Table 5. A synthesis of this complex is detailed in reference [66].

**Table 5:** Characteristics of **FeC1** from [66–68].

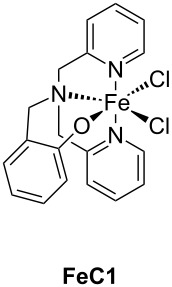		

		references

appearance	solid	
transitions observed	MLCT transition : pπ → dσ* transition centered at 360 nm pπ → dπ* transition centered at 590 nm	[66]
nature of the excited state	triplet	[66]
lifetime of the excited state	>1 ns	[67]
reduction potential	*E*_1/2_^red^ = −1.57 V	[68]

This iron complex with tetradentate monophenolate ligands has raised interest for catalytic reduction of hydrogen gas [64]. Such an iron polypyridyl complex has also really good photoredox catalyst properties to initiate a polymerization upon sunlight exposure in a three-component system [68]. Functionalization of the ligand can change the photochemical properties of the complex as described in [68], i.e., nitro-functionalization and sulfino-functionalization decreased the photocatalytic activity of the complex. Moreover, this functionalization affects the oxidative quenching rate and the stability of the complex. Thus, as for other complexes described above, the choice of the ligand is essential for good properties.

**2.1.3 The latest generation of metal-based catalysts: Emergence of the TADF complexes:** Metal complexes are still at the origin of numerous researches as photoredox catalysts, these researches being notably motivated by their remarkable long-lived excited state lifetimes that make these structures highly reactive structures. Since 2012 and thanks to the pioneering works of Adachi et al. in this field [69–71], a new class of metal-based complexes has been developed for photoredox application: TADF (abbreviation for Thermally Activated Delayed Fluorescence) complexes.

These compounds have singular excited states: their HOMO and their LUMO have been designed to avoid the overlap of the frontier molecular orbitals [32,69]. The energy between the singlet excited state and the triplet excited state becomes inferior to 0.1 eV, an energy easily overcome by thermal activation. Thus, the excited states can be thermally upconverted to the singlet state by reverse intersystem crossing (RISC), giving rise to a luminescence process arising from the singlet state (fluorescence). The concept of delayed fluorescence is based on the unusual and transient localization of the electrons on the triplet state, which upconvert to the singlet state thermally (i.e., at room temperature) and can promote a radiative decay from the singlet excited state. This property gives to TADF complex lifetime of their excited state comparable to the lifetime of the excited state of phosphorescent molecules, e.g., in the microsecond time scale [69].

Due to these really interesting properties, metal-based and metal-free TADF fluorescent materials have been extensively studied over the past few years, improving the photophysics of this new class of materials by molecular design [72–74]. This year, the first use of TADF complexes as photoredox catalysts in polymerization was reported [75]. **CuC 4** whose properties are presented in Table 6 is one of them. The synthesis of this complex has been reported in ref [58]. Other TADF copper complexes which can be used in photoredox cycles are described in this reference.

By their broad absorptions extending from 350 to 450 nm, theses complexes are excellent candidates for photoinitiation in the visible range. Moreover, the reactivity of the chromophores can be tuned by the counter anion, PF_6_^−^ being less nucleophilic than BF_4_^−^. In this context, both the polymerization rate and the final conversion were both improved by reducing the nucleophilicity of the anion in cationic polymerization. The photochemical properties of TADF **CuC 4** are summarized in Table 6.

**Table 6:** Characteristics of TADF **CuC 4** from [58].

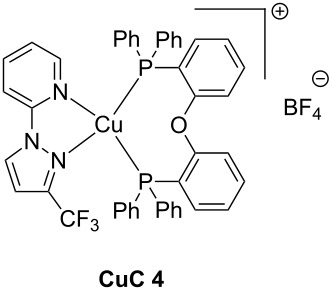	

appearance	solid
transition observed	MLCT transition (strong absorption at 355 nm)
nature of the excited state	triplet
lifetime of the excited state	2500 ns
oxidation potential	*E*_1/2_^ox^ = +1.42 V

From Table 6, it is observable that the properties of **CuC 4** are compatible with its use in a photoredox cycle with similar reactions than Ru-based complexes. Indeed, MLCT is also observed in the near-UV range to reach a triplet excited state with a long lifetime and oxidation potential is compatible with additives involved into a photocatalytical cycle.

#### 2.2 The organophotoredox catalysts

For some specific applications, it can be essential to develop metal-free systems because of potential toxicity, storage stability or bioaccumulation of metal for example. Organic photoredox catalysis has been largely studied in the past few years and is the topic of many reviews [17,40,45,49,52]. Thanks to the ability of some chromophores to transfer electrons when irradiated, they can participate in catalytic processes. These later catalytic processes can be widely applied to organic synthesis or photopolymerization [17]. More than just a “metal-free” alternative, some chromophores allow access to unique chemistries such as photocatalysis at different wavelength or photoconductivity for example [76]. Moreover, they can be characterized by lower costs. For examples, methylene blue or eosin-Y are examples of widespread photoredox catalytic dyes [18].

**Scheme 4:**
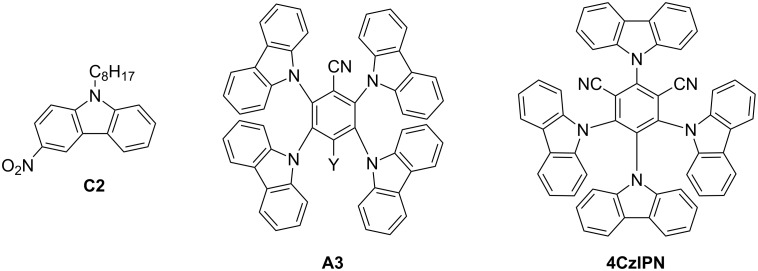
Structures of photoredox organocatalysts.

There is a wide range of organophotoredox catalysts. Here, only two examples of photoredox catalysts will be detailed: the carbazole derivatives and the TADF compounds presented as the last generation of organophotoredox catalysts. These examples are depicted in Scheme 4.

**2.2.1 Carbazole derivatives:** Carbazole derivatives are a good example of organophotoredox catalysts. A carbazole is an aromatic tricyclic organic compound with two six-membered benzene rings fused on either side of a five-membered nitrogen-containing ring. They exhibit unusual optical and electronic properties such as photoconductivity and photorefractivity [77]. More particularly, they are interesting for their high triplet energies, their ability to be quenched by either an electron donor or acceptor, and their reversible oxidation processes [78–79]. To finish, these compounds absorb in the near UV or visible range and related extinction coefficients are found relatively high. Thus, carbazole and its derivatives are good candidates for photoredox catalytic applications.

As an illustration, **C2** (abbreviation for 3-nitro-9-octyl-9*H*-carbazole) has been chosen and is depicted in Scheme 4. This photoredox catalyst can be synthetized as presented in [81]; its photochemical properties are given in Table 7.

**Table 7:** Characteristics of **C2** from [80].

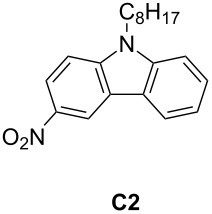	

appearance	powder
transition observed	π → π* transition centered at 374 nm
nature of the excited state	triplet
lifetime of the excited state	10 µs under nitrogen and 330 ns under air
oxidation potential reduction potential	*E*_1/2_^ox^ = +1.5 V *E*_1/2_^red^ = −1.38 V

We observe from Table 7 that by irradiation in the near-UV range, the excited state of **C2** is reached. This phenomenon can be observed upon exposure to different light irradiation such as light emitting diodes (LEDs) from 405 to 477 nm or a household device for example [80]. The triplet excited state is obtained. From the triplet state of carbazole, similar reactions with ruthenium triplet state (reactions 1, 2, and 3 in Section 2.1.1) can be observed. Moreover, as both oxidation and reduction potential are compatible with additives presented in Scheme 1, both oxidative photocatalytical cycle and reductive photocatalytical cycle are observed with this type of compounds [80]. This is possible only with an appropriate lifetime of the excited state which is remarkably high as we can see in Table 7.

**2.2.2 Emergence of the TADF carbazole derivatives:** TADF compounds as good candidates for photoredox catalysis have already been presented in Section 2.1.3. It is also possible to find in the literature metal-free TADF compounds with successful application in the photoredox catalysis field [81]. With adequate substituents on the carbazole structures described above, this thermally activated delayed fluorescence property has been observed combined with photoredox catalytical behaviour from UV to 450 nm light.

**A3** (abbreviation for 2,3,5,6-tetrakis(*N*-carbazolyl)benzonitrile) is one of those compounds (Scheme 4). A synthetic pathway of **A3** has been detailed in reference [81]. Its absorption extends from UV until 450 nm and can thus be activated at 405 nm with a blue light. Eventually, the peripheral carbazoles can be substituted with halogens. This increases the rate of RISC, and red-shifts the absorption spectrum [81]. For example, with a bromo atom on the peripheral carbazole of **A3**, the absorption at 470 nm is around 800 M^−1^·cm^−1^ where for **A3** almost no absorption is observed. Main characteristics of **A3** have been resumed in Table 8.

**Table 8:** Characteristics of **A3** [81].

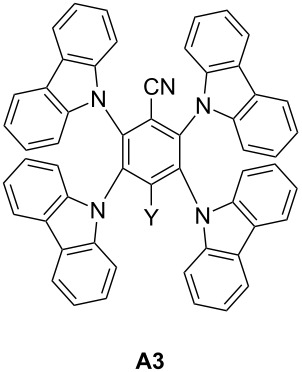	

appearance	powder
transition observed	π → π* transition at 333 nm
nature of the excited state	both singlet and triplet
lifetime of the singlet excited state	7.24 µs under nitrogen and 350 ns under air for both states (TADF properties)
oxidation potential reduction potential	*E*_1/2_^ox^ = +1.61 V *E*_1/2_^red^ = −1.63 V

The **A3** component can be used both as electron donor and electron acceptor. Indeed, the free energy change (calculated with the Rehm–Weller equation and the redox potential in Table 8) for the electron-transfer reaction is possible both with electron acceptor such as iodonium salt and electron donor such as EDB [76]. In a three-component system **A3**/Iod/EDB (where Iod stands for bis(4-*tert*-butylphenyl)iodonium hexafluorophosphate), both oxidative and reductive cycle can be observed and the two routes are even in competition and occur simultaneously. This is reinforced by remarkably long lifetime excited state (Table 8).

In organic chemistry, TADF molecules can also be used as organocatalysts for classical organic reactions that are traditionally carried out in the presence of transition metal complexes. In this field, **4CzIPN** (Scheme 4) that was reported in the first work of Adachi et al. as a green emitter for OLEDs [69,83–84] was revisited numerous times in organocatalysis. In 2017, it was notably used for the chemoselective and regioselective hydroformylation of aromatic olefins [84]. Interestingly, if transition metals can initiate an ionic hydroformylation reaction of aryl olefins, a free radical pathway could be promoted by use of diethoxyacetic acid and **4CzIPN**, inducing the in situ generation of an equivalent of a formyl radical.

Parallel to this, **4CzIPN** was also used as an organoredox catalyst for the alkylation of heteroarenes [85], the oxidation of silicates [86], the alkylation of imines [87], the α-arylation/heteroarylation of 2-trifluoroboratochromanones [88]. In these different situations, comparisons with reference transition metal catalysts classically used as initiators were established and **4CzIPN** could provide performances comparable to that obtained with metal complexes. As specificity, **4CzIPN** is characterized by a broad absorption extending from 250 nm to ≈500 nm. Its different photophysical characteristics are detailed in Table 9.

**Table 9:** Characteristics of **4CzIPN** [69,82–83].

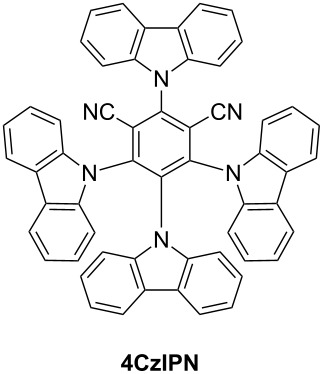		

		references

appearance	powder	
transition observed	π → π* transition around 290 nm	[69]
excited state	singlet and triplet	[82]
lifetime of the excited state	5.1 µs under nitrogen and 91 ns under air for both states (TADF)	[83]
oxidation potential	*E*_1/2_^ox^ = +1.35 V	[83]

**Scheme 5:**
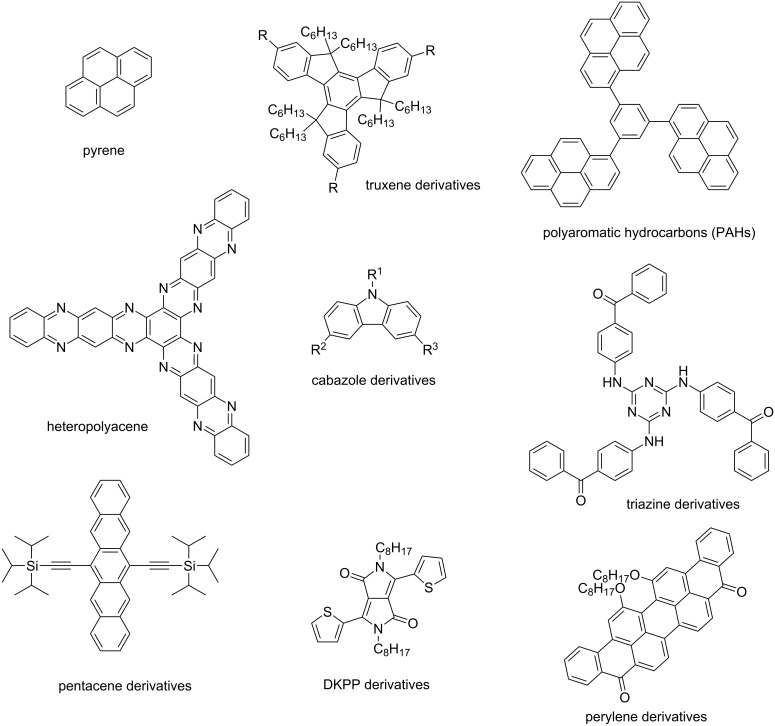
Diversity of the chemical structures of photoredox organocatalysts.

In order to be concise, only three kinds of organophotocatalysts have been described in the present review. However, over the years, a large number of different organophotocalysts has been described in the literature. Notably, pyrene [89], truxene [90–92], polyaromatic hydrocarbons [93], heteropolyacenes [90–91], carbazoles [80–81,94], triazines [95], pentacenes [96], diketopyrrolopyrroles [24] and perylene [97–98] derivatives have been investigated as organocatalysts as exemplified in Scheme 5.

### 3 Comparison of the efficiency of these photoredox catalysts in polymerization reactions

Polymerization reactions whose initiation is induced by photoredox catalysts has been detailed in part 1.4. Representative monomer conversions with different photoredox catalysts and upon different irradiation light for free radical polymerization and for cationic polymerization are, respectively, gathered in Table 10 and Table 11.

**Table 10:** Free radical polymerization performances with metal-based and metal-free photocatalysts [46,53,58,63,80–81,99].

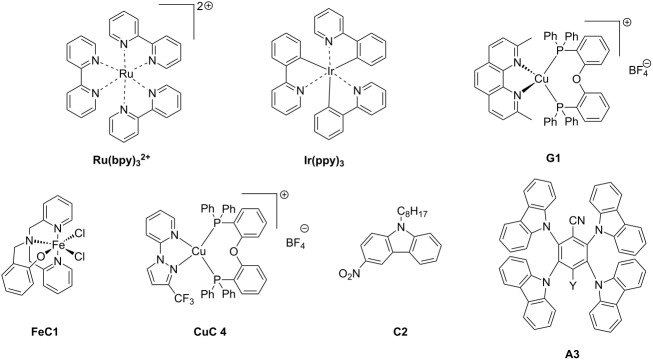						

photocatalyst	class of PC	monomer	irradiation	additives	conversion	references

**Ru(bpy)_3_^2+^** (0.2 wt %)	ruthenium complex	TMPTA	xenon lamp (λ > 390 nm)	Ph_2_I^+^ (2 wt %) (TMS)_3_SiH (3 wt %)	23% laminated 20 µm 200 s of irradiation	[46]
**Ir(ppy)_3_** (0.2 wt %)	irridium complex	TMPTA	xenon lamp (λ > 390 nm)	Ph_2_I^+^ (2 wt %) (TMS)_3_SiH (3 wt %)	40% laminated 25 µm 200 s of irradiation	[53]
**G1** (0.1 wt %)	copper complex	TMPTA	LED@455 nm (80 mW/cm²)	Ph_2_I^+^ (2 wt %) NVK (3 wt %)	56% laminated 20 µm 400 s of irradiation	[63]
**FeC1** (0.2 wt %)	iron complex	TMPTA	LED@405 nm (110 mW/cm²)	Ph_2_I^+^ (2 wt %) NVK (3 wt %)	31% laminated 20 µm 400 s of irradiation	[99]
**CuC 4** (0.5 wt %)	copper complex (TADF)	BisGMA/ TEGDMA	LED@405 nm (110 mW/cm²)	Ph_2_I^+^ (1 wt %) EDB (1 wt %)	80% under air 1.4 mm 800 s of irradiation	[58]
**C2** (0.5 wt %)	organic	TMPTA	LED@405 nm (110 W/cm²)	Ph_2_I^+^ (1 wt %) EDB (1.5 wt %)	57% laminated 25 µm 800 s of irradiation	[80]
		BisGMA/ TEGDMA	LED@477 nm (110 W/cm²)		44% laminated 25 µm 800 s of irradiation	[80]
**A3** (0.5 wt %)	organic	TMPTA	LED@405 nm (110 W/cm²)	Ph_2_I^+^ (1 wt %) EDB (1 wt %)	62% laminated 25 µm 800 s of irradiation	[81]

**Table 11:** Cationic photopolymerization performance with metal-based and metal-free photoredox catalysts [46,53,58,63,80–81,99–100].

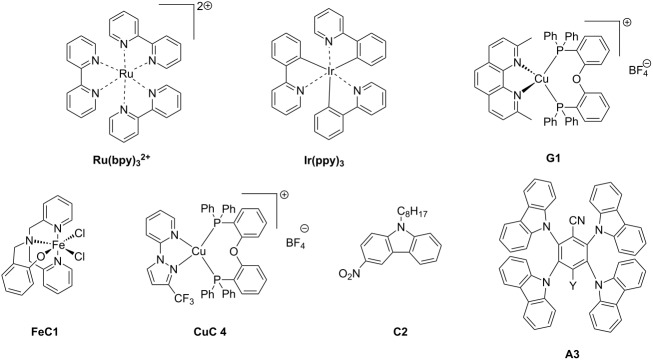						

photocatalyst	class of PC	monomer	irradiation	additives	conversion	references

**Ru(bpy)_3_^2+^** (2 wt %)	ruthenium complex	EPOX	laser diode @457 nm (100 mW/cm^2^)	Ph_2_I^+^ (2 wt %) (TMS)_3_SiH (3 wt %)	60% laminated 20 µm 200 s of irradiation	[46]
**Ir(ppy)_3_** (0.2 wt %)	irridium complex	EPOX	blue LED bulb (15 mW/cm²)	Ph_2_I^+^ (2 wt %) (TMS)_3_SiH (3 wt %)	63% laminated 25 µm 180 s of irradiation	[53]
**G1** (0.1 wt %)	copper complex	EPOX	LED@455 nm (80 mW/cm^2^)	Ph_2_I^+^ (2 wt %) NVK (3 wt %)	58% under air 20 µm 800 s of irradiation	[63]
**FeC1** (0.2 wt %)	iron complex	EPOX	LED@405 nm (110 mW/cm^2^)	Ph_2_I^+^ (2 wt %) NVK (3 wt %)	25% under air 20 µm 800 s of irradiation	[99]
**CuC 4** (0.5 wt %)	copper complex (TADF)	EPOX	LED@405 nm (110 mW/cm^2^)	Ph_2_I^+^ (1 wt %) CARET^a^ (1 wt %)	63% under air 1.4 mm 800 s of irradiation	[58]
**C2** (0.5 wt %)	organic	EPOX	LED@405 nm (110 mW/cm^2^)	Ph_2_I^+^ (1 wt %) EDB (1 wt %)	50% 1.4mm under air 800 s of irradiation	[80]
**A3** (0.5 wt %)	organic	EPOX	LED@405 nm (110 mW/cm^2^)	Ph_2_I^+^ (1 wt %) EDB (1 wt %)	54% 25 µm under air 800 s of irradiation	[81]

^a^See Scheme 7 below.

**Scheme 6:**
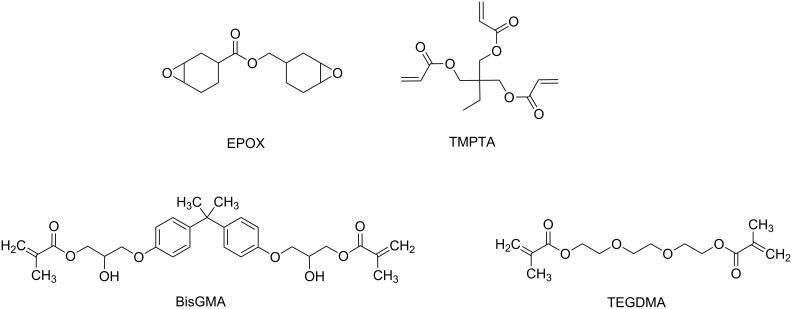
Structures of benchmarked monomers.

Additives used for both free radical polymerization and cationic polymerization are discussed in Section 1 and depicted in Scheme 1. Photocatalysts mentioned in Table 10 and Table 11 are the ones described in Section 2 and depicted in Scheme 2 and Scheme 4. To finish, monomers used as example for photopolymerization performance are shown in Scheme 6.

Two types of monomers are presented in Scheme 6. EPOX is a diepoxide which can be polymerized by cationic polymerization. Photopolymerization of the EPOX monomer can be followed by real-time Fourier transformation infrared spectroscopy, following the epoxy function. Other monomers presented in Scheme 6 are acrylates and methacrylates and can be polymerized by free radical polymerization. Photopolymerization of these compounds can also be characterized by FTIR measurement, following the C=C double bound conversion. The photopolymerization performance of the free radical polymerization using photoredox catalysis is presented in Table 10 and for cationic polymerization in Table 11.

As observed in Table 10, all photocatalysts presented before lead to a free radical polymerization. The first interesting property of a photopolymerization using a photoredox catalyst is the percentage of photoredox catalyst used. Actually, we observed that all polymerizations are performed using less than 0.5 wt % of photoredox catalyst. In most cases, the photoredox catalyst is the most expensive compound of the formulation and using a catalytic system instead of “traditional” PIs, can drastically reduce the final price of the system. Secondly, we noticed that there is no notably difference between the reactivities using a metal-based and a metal-free photoredox catalyst. The choice of the photocatalyst has to be done regarding the application. We observe that different types of light sources are used depending on the system.

For metal-based photocatalysts, the TADF derivative presented gives a remarkably high rate of final conversion for TMPTA. Moreover, the photopolymerization is done under air whereas other photopolymerizations are conducted in a laminated environment. This polymerization is really challenging due to the oxygen inhibition which prove the great efficiency of the system. The organobased photoredox catalyst **A3** gives also a really interesting final conversion.

**Scheme 7:**
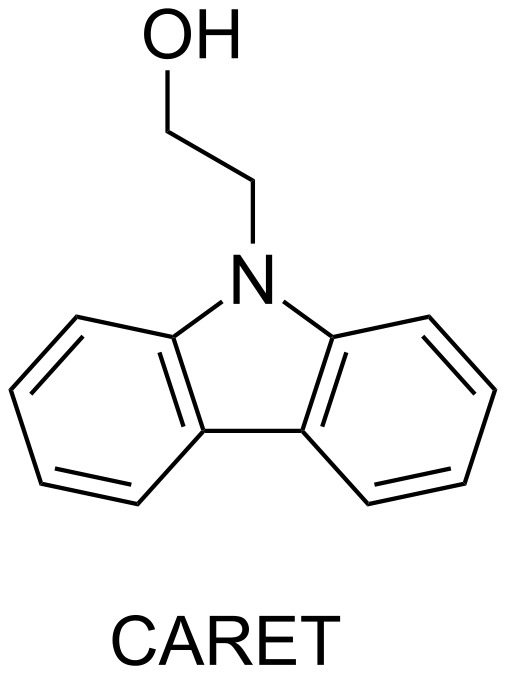
Structure of the CARET additive.

The **CuC 4** photoredox catalyst has been described in the literature using the CARET additive for photopolymerization of EPOX. CARET is depicted in Scheme 7.

This compound has exactly the same mechanism of reaction than EDB. There is first a hydrogen abstraction on the compound followed by a reaction with the iodonium salt into the photoredox catalytic cycle as described in Part 1.4, Figure 5.

Regarding the photopolymerization performances reported in Table 11, similar remarks can be made. Photopolymerization of cationic monomer is possible using only a catalytic content of PC and both metal-based and metal-free PC gives polymerization under the conditions tested. The choice of the photoredox catalyst has also to be done regarding the application: final toxicity, choice of the device to perform the polymerization (light irradiation, under air or not…), price of the formulation, etc.

For both free radical and cationic polymerizations, only a very small part of the wide diversity of possible photoredox catalyst has been presented here. Moreover, wavelength of irradiation is here restricted from 300 to 500 nm. In the recent literature, free radical polymerization using a photoredox catalyst has for example been presented using NIR light with both metal-based and metal-free photoredox catalyst [101].

### 4 Photoredox catalysts in controlled radical polymerization

Photoredox catalysis can also be used in a controlled free radical polymerization. The controlled radical polymerization is a powerful tool for the synthesis of polymers with precise average molar masses, diverse compositions and well-defined architectures [102]. The controlled radical polymerization has raised lots of interest and allows high chain end fidelity and ability to reinitiate the polymer chain.

The use of light to mediate radical photopolymerization can influence two different processes: intramolecular photochemical processes and/or photoredox processes. In this review, we will focus only on photoredox processes. In a photoredox-controlled radical polymerization, a photoredox catalyst is used. By irradiation, it undergoes a single electron transfer with an appropriate initiator. Thus, radicals are produced to initiate the polymerization. The polymerization can be controlled through oxidative or reductive pathways.

**Scheme 8:**
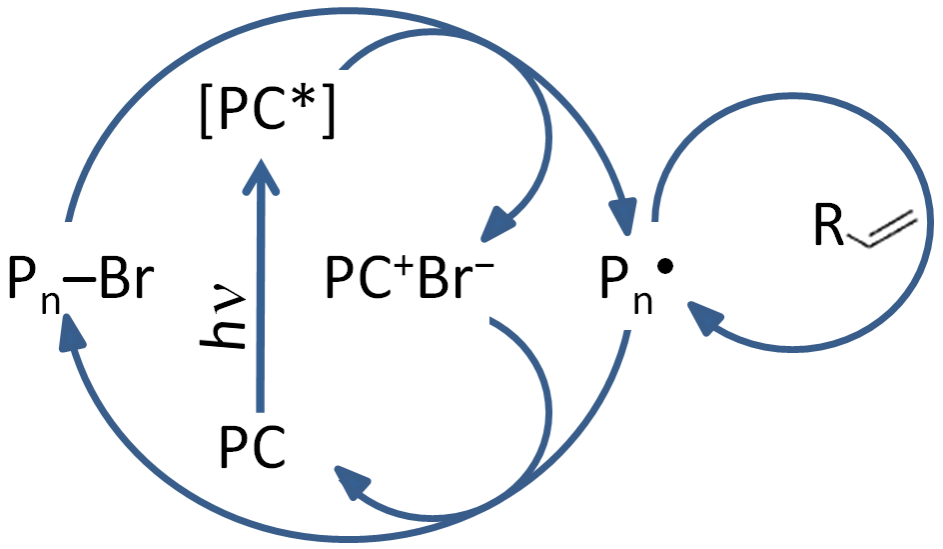
Photoredox catalysis mechanism of a visible light-mediated living radical polymerization. (Abbreviation: PC for photoredox catalyst, [PC]* the photoredox catalyst at its excited state, P_n_ the polymeric chain), extracted from [103].

An example is provided where the controlled photopolymerizations are based on an oxidative quenching mechanism. This means that the photoredox catalyst is irradiated to go to its excited state and then oxidized by the initiator or the dormant species (R-Mn-Br) [102]. To regenerate the PC, a single electron transfer reaction must be involved as shown in Scheme 8.

Through these single electron transfer processes, photo-ATRP has been successfully achieved (ATRP stands for atom transfer radical polymerization) [104–105]. It is defined by IUPAC by “Controlled reversible-deactivation radical polymerization in which the deactivation of the radicals involves reversible atom transfer or reversible group transfer catalyzed usually, though not exclusively, by transition metal complexes” [106]. In this mechanism, the catalyst is the most important component: it determines the equilibrium constant between the active and dormant species which is directly linked to the distribution of chain lengths [107].

As photoredox catalysts for ATRP applications, copper(II)-complexes have been widely used. For example, bis(1,10-phenanthroline)copper(I) (abbreviated Cu(phen)_2_^2+^) is reported in ref [37] as an efficient photoredox catalyst for ATRP upon a simple household blue LED. Ir-based photoredox catalyst are also efficient in this mechanism such as tris(2-phenylpyridinato)iridium(III) reported in ref [37] for photocontrolled radical polymerization of methacrylates. The use of a Fe complex or dye (for metal-free PC) is also possible to performed ATRP as described in reference [107].

A ROMP mechanism is also possible thanks to the photoredox catalyst. ROMP stands for ring-opening metathesis polymerization. This polymerization is based on cyclic olefins whose ring strain is released during polymerization. This reaction needs a catalyst to occur [108]. Metal-free photoredox catalysts for ROMP were proposed in [109]. For example, pyrylium and acridinium salts can be used as good candidates for photooxidation of vinyl ether initiators. A Ru-based ROMP precatalyst: [Ru(IMesH_2_)(CF_3_CO_2_)(*t-*BuCN)_4_)]^+^ CF_3_CO_2_^–^ which is a thermally stable photoredox catalyst has been also proposed in [110]. To finish, the RAFT polymerization can be also extended in a photocontrolled polymerization accessible via a photoredox catalyst. RAFT is the abbreviation of reversible addition-fragmentation chain transfer. A RAFT agent is necessary to perform the polymerization [111]. However, ruthenium or iridium PCs have been used in RAFT polymerizations through a photoredox catalytic cycle to suppress oxygen inhibition [112]. Organic photoredox catalysts can also be used to perform a RAFT polymerization, i.e., in [113] trithiocarbonates were proposed as intrinsic photoredox catalysts and RAFT agents.

## Conclusion

In the present paper, some examples of metal and metal-free photoredox catalysts are provided for photoreticulation processes of multifunctional radical (acrylate, methacrylate…) or cationic (epoxide) monomers. Undoubtedly, the advantage of the photoredox catalysis approach is the high efficiency of the system to initiate the polymerization upon mild light irradiation conditions (as the catalyst is regenerated) and the low content required. All these works on PC pave the way for highly reactive photosensitive systems that can be used for high tech applications: functional coatings, smart materials, new 3D printing resins, preparation of composites. Other development of PC can be expected in near future.
